# Doping porous silicon with erbium: pores filling as a method to limit the Er-clustering effects and increasing its light emission

**DOI:** 10.1038/s41598-017-06567-4

**Published:** 2017-07-20

**Authors:** Guido Mula, Tony Printemps, Christophe Licitra, Elisa Sogne, Francesco D’Acapito, Narciso Gambacorti, Nicola Sestu, Michele Saba, Elisa Pinna, Daniele Chiriu, Pier Carlo Ricci, Alberto Casu, Francesco Quochi, Andrea Mura, Giovanni Bongiovanni, Andrea Falqui

**Affiliations:** 1 Dipartimento di Fisica, Cittadella Universitaria di Monserrato, Università degli Studi di Cagliari, S.P. 8 km 0.700, 09042 Monserrato (Ca), Italy; 2 CNR-IOM - Istituto Officina dei Materiali c/o Laboratorio Materiali Porosi, Dipartimento di Fisica, Università degli Studi di Cagliari, Cittadella Universitaria di Monserrato, S.P. 8, km 0.700, 09042 Monserrato (Ca), Italy; 3 Université Grenoble Alpes, F-38000 Grenoble, France; 4 CEA, LETI, MINATEC Campus, F-38054 Grenoble, France; 5 King Abdullah University of Science and Technology (KAUST), Biological and Environmental Sciences and Engineering (BESE) Division, Nabla Lab, Thuwal 23955-6900 Saudi Arabia; 6 CNR-IOM-OGG c/o ESRF, LISA CRG, 71 Av. des Martyrs, F-38043 Grenoble, France

## Abstract

Er clustering plays a major role in hindering sufficient optical gain in Er-doped Si materials. For porous Si, the long-standing failure to govern the clustering has been attributed to insufficient knowledge of the several, concomitant and complex processes occurring during the electrochemical Er-doping. We propose here an alternative road to solve the issue: instead of looking for an equilibrium between Er content and light emission using 1–2% Er, we propose to significantly increase the electrochemical doping level to reach the filling the porous silicon pores with luminescent Er-rich material. To better understand the intricate and superposing phenomena of this process, we exploit an original approach based on needle electron tomography, EXAFS and photoluminescence. Needle electron tomography surprisingly shows a heterogeneous distribution of Er content in the silicon thin pores that until now couldn’t be revealed by the sole use of scanning electron microscopy compositional mapping. Besides, while showing that pore filling leads to enhanced photoluminescence emission, we demonstrate that the latter is originated from both erbium oxide and silicate. These results give a much deeper understanding of the photoluminescence origin down to nanoscale and could lead to novel approaches focused on noteworthy enhancement of Er-related photoluminescence in porous silicon.

## Introduction

The lack of significant photoluminescence (PL) from Si is due to its indirect bandgap and the several efforts made to overcome this limitation have resulted in hybrid, complex and expensive technological solutions^1^. As a consequence, the demand for alternative, simpler and cheaper fabrication processes of luminescent silicon has constantly grown. At the beginning of the nineties of last century, the quantum confinement effect on its crystalline structure made porous Si (PSi) an interesting candidate for light emission^2, 3^, but the complexity of the mechanisms originating the PL from nanostructured Si^4^ has until now made PSi ineffective for all-Si light emitting devices in view of technological applications, given the great difficulty in finding a unique identification of the PL origin in PSi. A second possible strategy was found in the rare earth doping of PSi as a mean to obtain efficient emission of light with wavelength λ = 1.5 µm^5–11^, as witnessed by the numerous works published on Er^3+^-related PL^6, 12–14^ or electroluminescence^15, 16^ from PSi. Such a high interest in the PL from Er^3+^ comes from the fact that its wavelength falls in the middle of most transparent transmission λ-window of silica optical fibers^17^.

This peculiar erbium PL resulted from electron intra-4*f* transitions of the incomplete shell, which, despite being forbidden in the free ion state, are partially allowed when Er^3+^ is hosted in a non-centrosymmetric site^18–20^. Er-Yb codoping gave better results than the sole Er doping for the PSi matrix^21, 22^, but despite all the efforts made in the rare earth doping of Si structures^23, 24^, no way has been found so far for obtaining light emission with a high enough yield^25, 26^. Er clustering surely plays an important role when trying to increase the availability of Er luminescent centers, and a lot of research effort has been spent on Silicon Rich Oxide (SRO) structures^27–34^, but the clustering is such a hard limiting factor for light emission from these materials that some authors already started to propose to lower Er doping levels^25^ to overcome the clustering effect even if it implies reducing the maximum achievable PL emission power.

The use of PSi as a hosting matrix implies a completely different process with respect to SRO structures, where the Er doping is mainly obtained by Er ion implanting. In particular, the possibility of using an electrochemical process for the doping of the structure means that the deposition of the Er^3+^ ions depends on the several control parameters of an electrochemical doping process, e.g. the solution concentration, the choice of constant current or voltage, the value of the current or voltage, the duration of the process^9, 35–38^. Since the impossibility to achieve a higher light emission yield from PSi has not been confirmed by any theoretical study, this long-standing failure had to be ascribed to the lasting lack of a satisfactory, detailed characterization of the actual electrochemical Er doping (ED) process^35–38^, without which an appropriate control of the Er deposition is impossible. In fact, even recent studies^14^ were focused on the optical yield in the silicon structures, both rich in silicon oxide^13^ or made by PSi^7, 12^. However, both the large surface area of PSi and its dendritic structure make understanding and controlling the ED process a not trivial, relatively uncharted task^35–39^. In recent studies from some of the coauthors, several and concomitant aspects of the ED were presented, revealing the complexity of this matter: a) the Er content and refractive index variation are not linearly dependent on the current intensity and density^38^; b) at least two different electrochemical processes occur during the doping, one of which seeming to be activated only for higher currents^35^; c) a gradient of Er content in the porous layer depth is always observed, and it depends on the doping current used^37^. Although the reported results gave some insights concerning how the ED process works, it appeared finally that the only way to really define new routes towards the PL enhancement relies on an improved, highly resolved characterization of the obtained materials from a structural and optical point of view, including the Er distribution within the porous structure. A high concentration of luminescent Er centers in the PSi matrix is needed to obtain high PL efficiency^40, 41^ and, since higher Er content implies higher Er clustering occurrence, the understanding of the doping process mechanism can help reducing the clustering drawbacks.

In this work, we propose to overcome the Er clustering limitation by changing the approach from the electrochemical Er doping (lower Er amount) to electrochemical Er infiltration (EI) to fill the silicon pores with Er-rich materials. In fact, literature reports as those from Miritello *et al*.^42, 43^ describe the behavior of two highly luminescent Er composites, Er silicate and Er oxide, that we propose to insert within the PSi matrix thanks to the EI, so that the PL emission can then be originated both from the Er within the solid PSi matrix and from the pores content.

To study this process and achieve a highly resolved 3D imaging of PSi:Er samples, we made use of Electron Tomography (ET) of samples prepared in needle-form (needle-ET) and spatially resolved micro-photoluminescence (µPL), together with the study of both surface and cross sectional erbium quantitative distribution by Energy Dispersive X-ray Spectrometry (EDS) *via* Scanning Electron Microscopy (SEM). Needle-ET^44^ is a quite novel and effective technique capable of providing a 3D reconstruction with nanometric resolution of the studied samples^45–47^, while µPL allows determining the photoluminescent surface areas with a spatial resolution of few microns. The use of time-resolved PL and Extended X-ray Absorption Fine Structure (EXAFS) allows then the individuation of the chemical compounds at the origin of the PL emission from the Er-infiltrated porous silicon samples. From the combination of these analytical tools we are able to reconstruct the 3D morphology and, at the same time, spatially resolve the chemical composition at the nanoscale of the samples regions where the Er atoms accumulate and fluoresce. This information leads then to gaining a so far missing and fundamental tile in comprehending the mechanisms governing the ED process and the related PL properties.

## Results

The experimental investigation here reported aimed at obtaining a wide overview of the samples features and related properties, taking into careful account the relations among the different preparation steps. First, we describe how the PSi preparation and the EI protocols are accomplished. Then, following the thermal treatment performed under N_2_ atmosphere to activate the Er-related photoluminescence, dehydrate and evaporate the carbon-related groups^48^, the results obtained by the investigation by PL and µPL, SEM imaging and related EDS, and needle-ET are shown, discussed and put in relation with the results expected by the different electrochemical doping process parameters.

### Samples fabrication and doping

The PSi samples were prepared with standard electrochemical etch in the dark of heavily *n*-doped Si wafers with resistivity in the 3–7 mΩ^.^cm in a HF:H_2_O:EtOH solution in the 15:15:70 proportion, respectively. This allows the formation of columnar dendritic pores with an average diameter of 10 nm. Although highly doped Si substrates suffer from strong free-carrier absorption^49^, in the case of porous silicon such an effect is strongly inhibited by the presence of surface states that confine the free carriers^50, 51^, thus remaining a problem mainly for ultrafast spectroscopy studies, where the high density of the optical pumps generates a large amount of free carriers^52^. For this reason, even highly doped substrates have been successfully used in the past for Er-doped PSi structures^53^. The electrochemical doping procedure, described in details elsewhere^38^, has been performed in constant-current regime in all our PSi layers. More details about the doping procedure will be given in the discussion section. All samples have 55% porosity, average pore diameter of 10–20 nm and 1.3 µm thickness.

**Figure 1:**
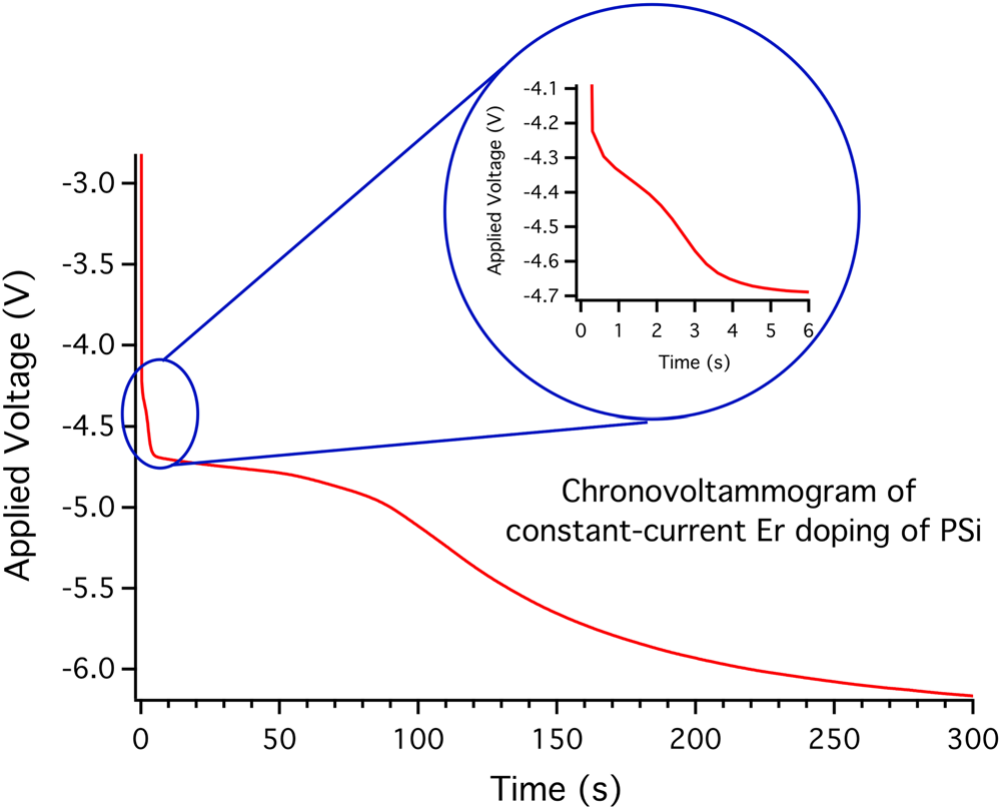
Evolution of the applied voltage during EI of PSi. A typical evolution of the voltage during a constant-current EI process for the insertion of Er into the PSi matrix is shown. The presence of several phases is evidenced by its non-constant behavior. In the inset, an enlargement of the double transient observed in the initial phases of the infiltration process is shown.

Figure 1 shows the typical behavior of a PSi sample that underwent an EI process for 300 s in constant-current conditions. The electrochemical behavior shows several features with clear slope changes depending on the chosen constant value of current^35^, which controls the activation of different processes during EI. In this work we only used the high-current regime, since it is the most efficient one in terms of accumulated Er per unit-transferred charge. This behavior is observed for all PSi samples, as confirmed by the very high similarity of the V(t) graphs.

As previously described, a thermal treatment of the PSi layer is a mandatory step to activate the Er luminescence in both crystalline^54, 55^ and porous silicon layers^48, 56^. In the present case, we chose to perform 700 °C thermal treatments in N_2_, the most commonly used inert gas atmosphere. The choice of such annealing temperature was properly made with the aim to obtain an efficient Er activation without significantly changing the pores structure. Higher temperatures, while they can be more effective for SRO structures, in PSi case can induce strong structural changes of the solid PSi matrix^57^, which can in turn lead to the formation of a structure more similar to SRO than to infiltrated PSi. On the other hand, lower temperatures while better conserving the PSi matrix structure may be inefficient for the Er activation. A temperature of 700 °C was then chosen since, while it is relatively low for best PL performances of Er silicate and oxide^42, 43^, is expected to ensure a good preservation of the porous matrix^57^ and an easier comparison of the results for the purpose of understanding the pores filling mechanisms.

To evaluate the effects of thermal treatments on the Er-infiltrated PSi, PL measurements were performed in several spots of the PSi samples before and after the thermal annealing. The PL measurements were performed in the same regions before and after the thermal treatment for a direct comparison, showing an intensity increase of several orders of magnitude after the annealing, thus confirming its efficacy in the PL activation.

However, the understanding of the EI in PSi is far from straightforward and gaining more information about that requires several techniques that span from the electrochemical to the structural and optical characterizations. We then chose to study our samples as a function of the Er content in the porous matrix, from 1–2% up to about 12%, that is from the standard doping level to the one where the surface accumulation of the Er ethanolate gel leads to the formation of a deposit clearly visible by naked eye.

For clarity and immediateness, in this work the samples will be referred to with names indicating their treatment according to the following scheme: PSi_Er-content_Annealing-state. For instance, a sample that underwent an EI process for 200 s, which is corresponding to 8% Er content, and finally annealed will be indicated as PSi_8%_A, while a sample prepared with the same parameters without annealing will be named PSi_8%. The equivalence between the doping level and doping times for the samples studied in this work is given in Table 1.

**Table 1:** EI duration and Er concentration equivalence. Equivalence between Er doping time and doping level for 1.3 µm thick samples when using a constant doping current of 1 mA.

Er infiltration time (s)	Er concentration (%)
25	1
30	1.2
100	4
150	6
200	8
250	10
300	12

### Er distribution and quantification

To avoid any possible misinterpretation of the results, the Er distribution in the samples over the PSi layer’s surface and along its thickness were investigated by SEM imaging and related EDS elemental mapping and quantification, in both planar and cross sectional view^58^. The results, shown in the Supplementary materials (Figs S1 and S2), ensure that Er is homogeneously distributed along the surface and shows, similarly to what previously observed by Mula *et al*.^35^, a decreasing gradient from the external surface towards the PSi/Si interface.

### Room temperature Photoluminescence

**Figure 2:**
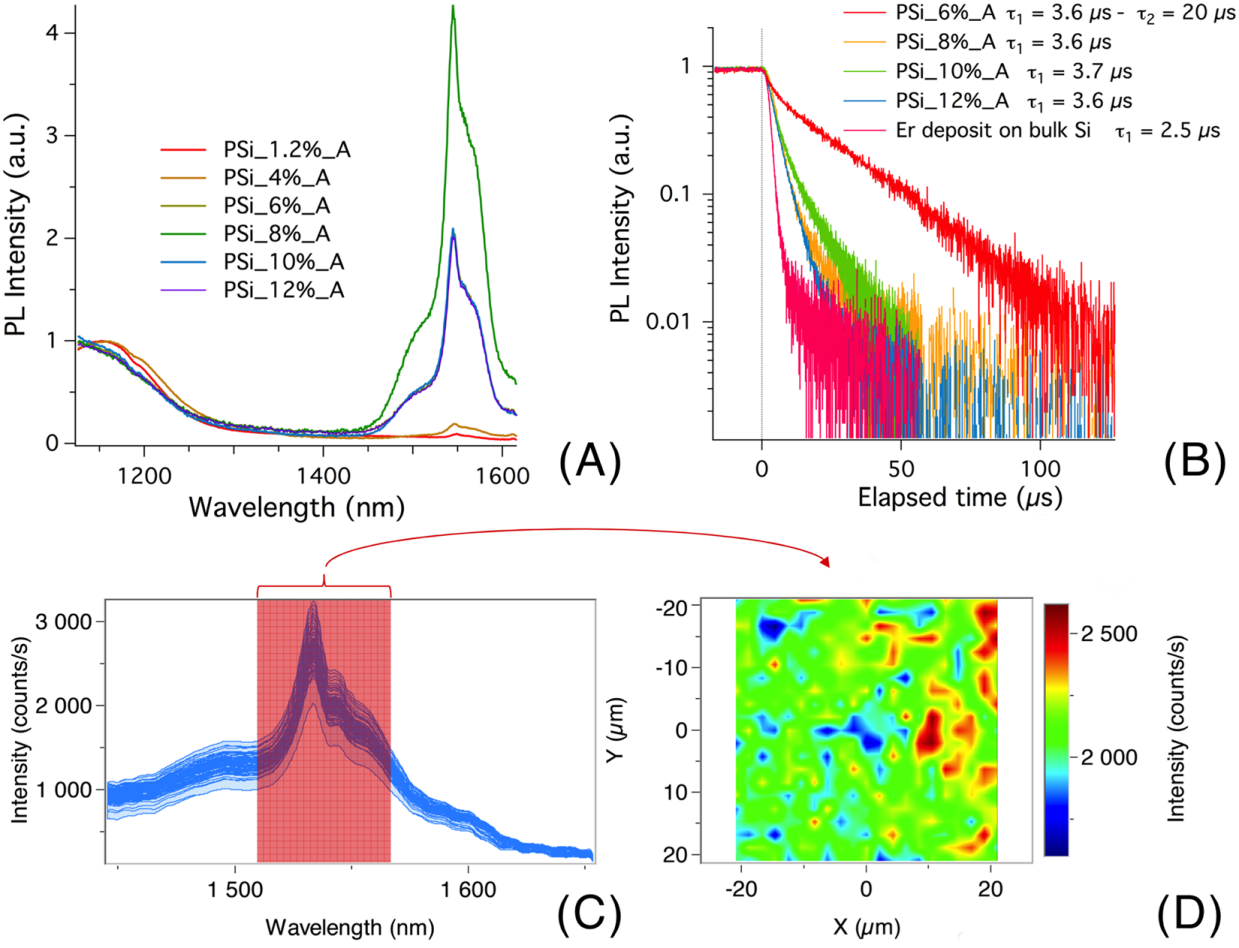
Photoluminescence results. (**A**) Normalized PL from annealed PSi samples with different Er content. (**B**) Decay time for the Er PL as a function of the Er content. The purple line with the shorter decay time has been measured from a thick Er-rich layer deposited on top of a bulk Si wafer using the same solution and doping current used for the doping of the porous samples. The deposition time in that case was 2000 s. (**C**) µPL measurements of a PSi_8%_A. (**D**): bidimensional mapping of µPL results shown in (**C**): −20 to 20 µm, step: 2 µm, in the central area of the sample; acquisition time: 10 s (background corrected); the intensity of each point on the map has been taken as the integrated intensity over the red area in the corresponding spectrum.

In Fig. 2(A) we report the PL measurements as a function of the Er doping level for a series of samples with increasing Er content: PSi_1.2%_A, PSi_4%_A, PSi_6%_A, PSi_8%_A, PSi_10%_A and PSi_12%_A. The PL spectra are normalized with respect to the maximum intensity of the Si emission for wavelengths shorter than 1200 nm to provide an easier comparison, although the absolute measurements also roughly follow the same trend. The data show a significant increase in the PL intensity with increasing Er content up to a maximum, after which a slight reduction and saturation of emitted intensity is observed.

### Time resolved PL

To further explore the evolution of the EI process as a function of the final Er content, the PL decay times of samples having a PL significant emission (starting from PSi_6%_A) were measured. These measurements do not aim at studying the luminescence dynamics but at evidencing different optical properties of the composite material. The results are shown in Fig. 2(B). The analysis was started from sample PSi_6%_A (red curve), where two decay times were observed, obtained by fitting a biexponential decay curve to the data: a faster decay (τ_1_ = 3.6 µs) followed by a slower one (τ_2_ = 20 µs). The other samples show a single fast decay time almost identical to τ_1_ and are all at least partially covered by an Er-rich film^38^. The purple line, showing the fastest decay time, has been measured on a relatively thick Er deposit on a bulk Si sample obtained using the same solution and constant current value used for EI but adopting a longer process duration (2000 s). In this case the measured decay time is shorter than for the PSi samples and is τ = 2.5 µs. The time resolution of our set-up, measured as the apparent duration of a sub-picosecond laser pulse, was found to be around 1 ns.

### PL mapping

The spatial homogeneity of the emitted light was also evaluated by µPL. Such a technique allows establishing whether the light comes predominantly from few hotspots, while luminescence from most of the film is quenched, or if the distribution reflects a mostly homogeneous spatial distribution of optically active Er ions on a micrometric scale. The µPL maps (Fig. 2(C) and (D)) show relatively small intensity fluctuations around the average value, which are compatible with small local Er content fluctuations and small local surface roughness, but no evidence of the existence of hotspots.

### Needle Electron Tomography by STEM-HAADF

**Figure 3:**
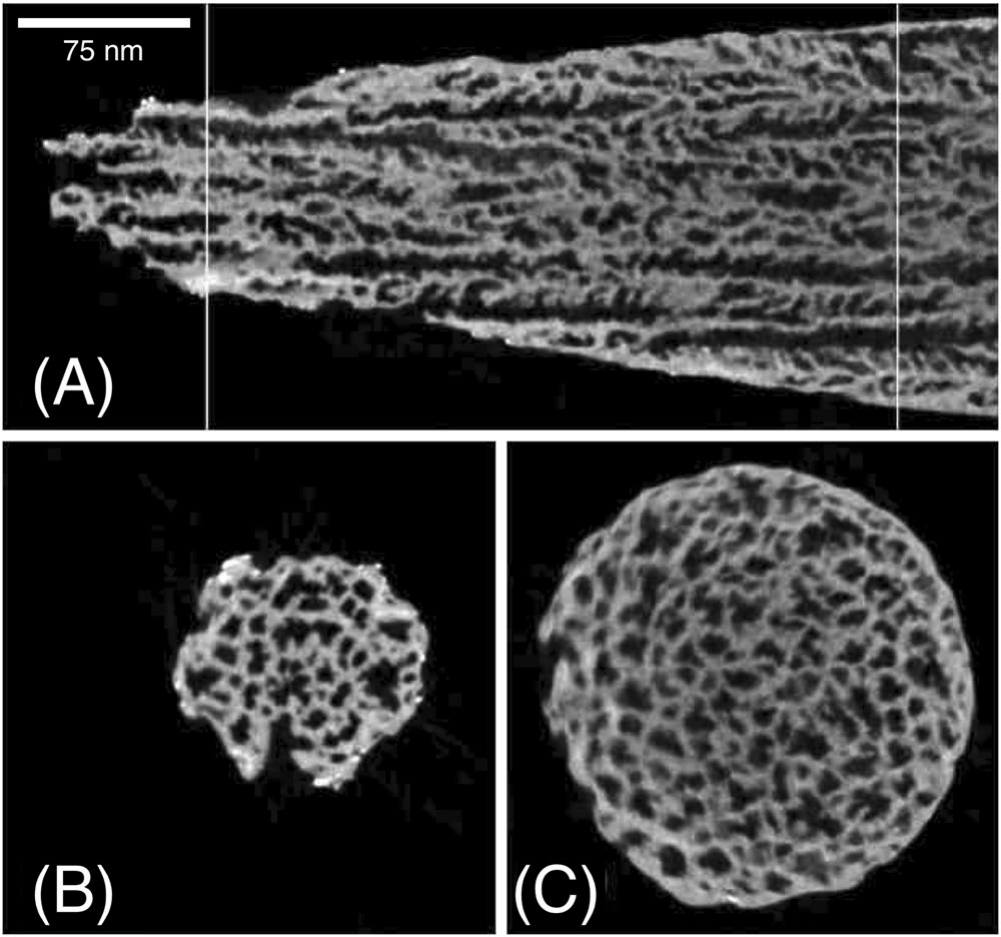
Needle-ET of the PSi_0 sample (pristine PSi sample). (**A**): sagittal sections extracted from the middle of the 3D reconstructed volume of the PSi needle, (**B**–**C**) axial sections extracted in correspondence of the white lines reported in panel (A).

**Figure 4:**
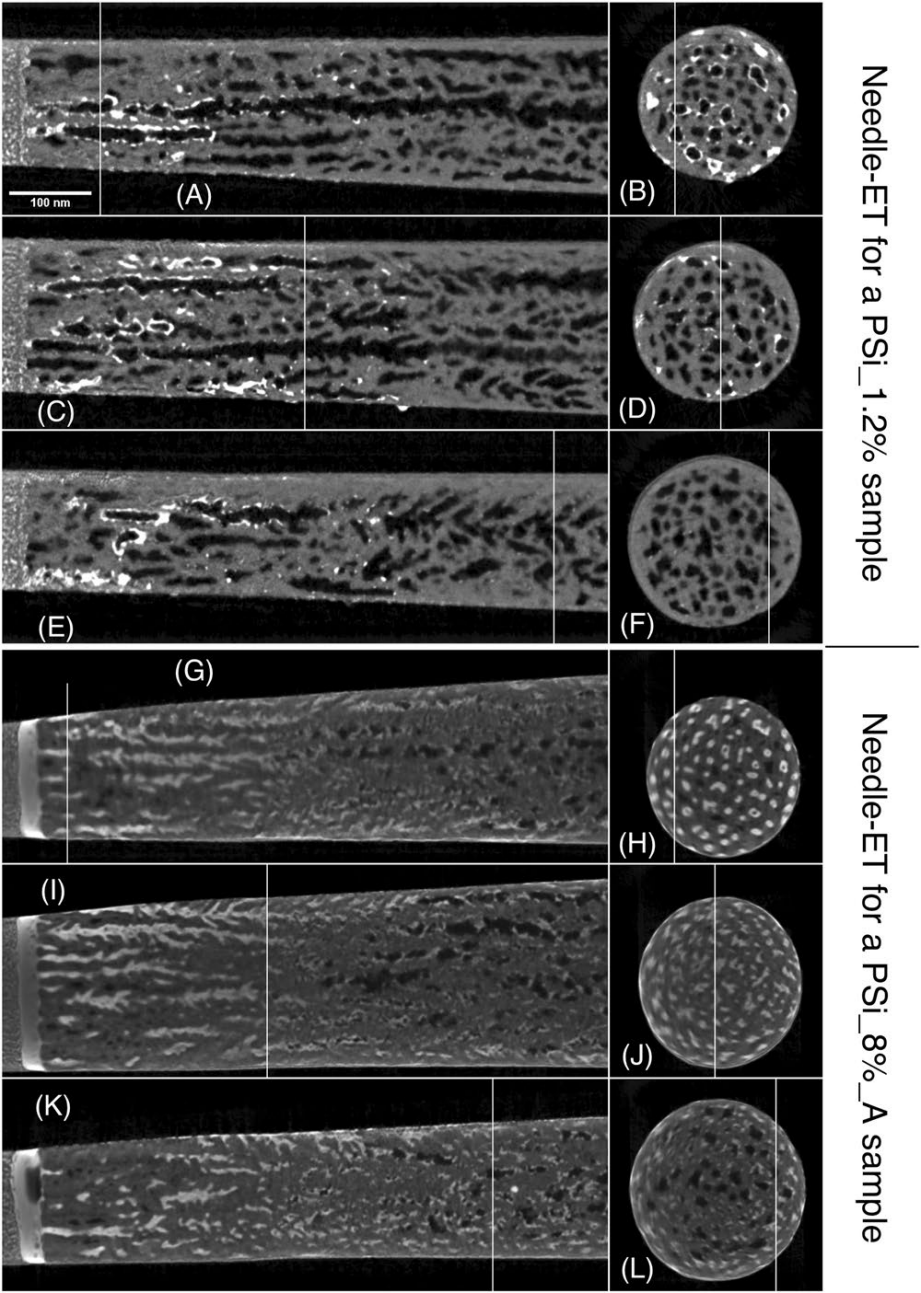
Needle-ET of PSi:Er samples. Panels (A) to (F) refer to a PSi_1.2% sample, while panels (**G**) to (**L)** refer to a PSi_8%_A sample. Panels (A), (C), (E), (G), (I) and (K) are different sagittal slices, while (**B**), (**D**), (**F**), (**H**), (**J**) and (**L**) are different axial slices in the 3D reconstructed volume. Localization of the sagittal slices are represented by a white line on the corresponding axial slices and vice versa. The surface of PSi specimen for both samples is on the left hand side of the figure. The surface of the original PSi specimen is visible on the extreme left part of the figure. The filling of the pores by the Er deposition is evidenced by the white signal from the PSi pores. A detailed description of the figure is in the text.

The needle-ET was performed in Scanning Transmission Electron Microscopy (STEM) mode, using a low energy acceleration voltage of 80 kV and a High Angular Annular Dark Field (HAADF) detector. The ensemble of these experimental parameters allows performing a STEM imaging with very high sensitivity to atomic number-based contrast (Z-contrast). This effect comes from the fact that atomically heavy elements give rise to much higher electron scattering than light elements. As a consequence, in the STEM-HAADF images the heavier elements give rise to brighter pixels than the lighter ones. Erbium (Z = 68) rich areas thus appear much brighter than silicon (Z = 14) parts or voids. Figures 3 and 4 show needle sections obtained from three different 3D volume reconstructions. Figure 3 shows the results on a reference PSi sample (PSi_0) with no Er-doping. Figure 4 refers to two Er-doped samples: a sample with a moderate Er-doping (PSi_1.2%) and one with high Er doping and subsequent thermal annealing (PSi_8%_A).

The needle-ET sections of PSi_0% sample, shown in Fig. 3, show a homogeneous distribution of pores from the surface to the more in-depth regions, towards the PSi/bulk Si interface. Very small, brighter clusters can be observed at the extreme borders of the needle-shaped sample, being them constituted by gallium as a well-known consequence of the FIB needle preparation.

The distribution of Er within the PSi matrix can be observed from the needle-ET sections of sample PSi_1.2% (Fig. 4, panels A-F). While a decreasing Er concentration gradient is observed from the surface towards the bulk Si, in agreement with the SEM-EDS measurements, a relevant distribution difference along the porous layer thickness is also found. Er distribution shows quite homogeneous nanometer-sized Er clusters on the pores inner surface far from the external surface (In Fig. S3, showing needle-ET of a PSi_1% sample, these clusters are more clearly visible for image contrast reasons) while towards the surface the clusters aggregate and cover the pores walls (the almost continuous white signal). It is worth noticing that not all of the pores show such an Er coverage: while most pores show a high Er content, some pores appear empty. This behavior is similar to that observed for electrochemically Ni- or Fe_3_O_4_-filled PSi samples^59, 60^, where not all the pores were filled. The pores filling process shows a high homogeneity on the porous layer (Supplementary materials, Fig. S2).

When further increasing the Er doping level, needle-ET of a PSi_8%_A sample (Fig. 4 panels G-L) displays an almost complete filling of the pores in the region close to the external surface, with some pores still empty (as for PSi_1.2%, Fig. 4 panels A–F) and the maximum depth for a complete pore filling being around 800 nm from the external surface. It can also be noticed that the shape of the filled pores remains unmodified with respect to the one observed before the annealing (e.g. PSi_1.2%, Fig. 4 panels A-F). The presence of Er accumulation at the sample surface is also apparent. Finally, in the sagittal views the filled pores display a dark core in their center, strongly suggesting that the pore filling is a process that starts from the pores surface and proceeds towards their central part.

### EXAFS measurements

**Figure 5:**
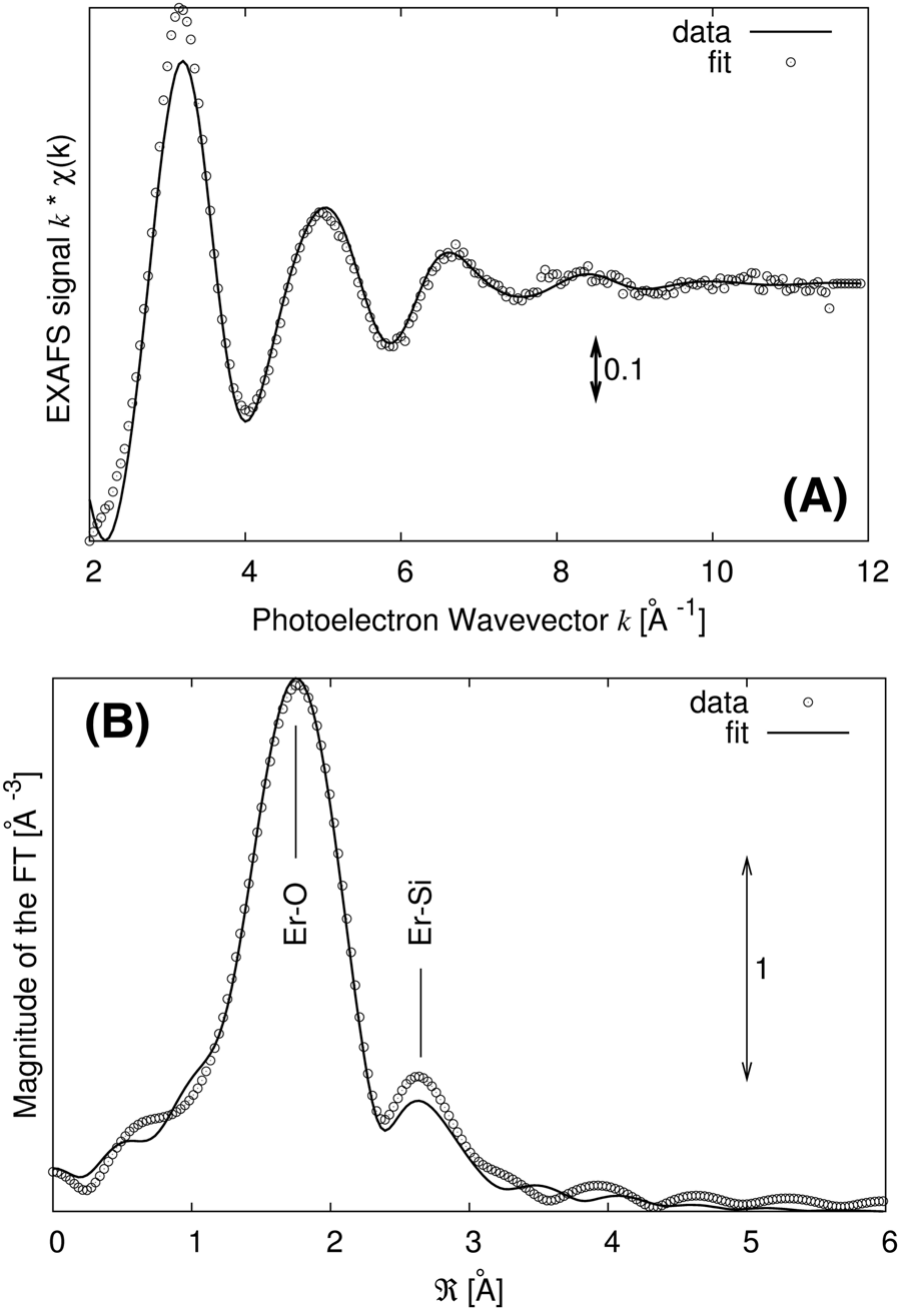
EXAFS measurements of a typical PSi_8%_A sample. (**A**) EXAFS spectrum and best fitting curve. (**B**) Fourier Transform of the EXAFS spectrum with the peak identification Er-O and Er-Si. Transforms were carried out in the interval K = 2.5–8 Å^−1^ with a k^2^ weighting factor.

The local Er environment has been studied by EXAFS experiments, whose data are presented in Fig. 5(A), whereas the related Fourier Transforms are presented in Fig. 5(B), for a typical PSi_8%_A sample.

The EXAFS spectrum is dominated by a single oscillation at low frequency, typical of light backscatterers (O, namely). The Fourier Transforms present a main peak with a shoulder on its right side as already reported for Er inserted in glassy matrices^61, 62^. The data analysis has been carried out using a 2-shell model: an Er-O first shell and an Er-Si second shell. This approach follows the idea that each O neighbor belongs to a SiO_4_ tetrahedron. Multiple scattering contributions were taken into account as reported by d’Acapito *et al*.^61, 62^. The results of the quantitative analysis are shown in Table 2.

**Table 2:** Results of the quantitative EXAFS analysis. The errors on the last figure are indicated in parentheses.

Sample	N_O_ = N_Si_	R_O_ (Å)	σ^2^_O_(Å^2^)	R_Si_ (Å)	σ^2^_Si_(Å^2^)
**PSi_8%_A**	8(5)	2.28(2)	0.015(5)	3.56(5)	0.03(1)

The Er-O distance is typical of highly coordinated Er whereas the Er-Si distance is typical of bonds bridged by an O atom^62^. No presence of Er-Si distances typical of silicides (R_ErSi_ 2.9–3.0 Å)^63^ is observed. The first and second shell bond distances are reported to impact the luminescence as reported^64–66^ and are related to the distortion of the Er environment, that enhances the intra-f transitions. The Er-O-Si angle is 133 deg, well in the 130–140 deg range reported in literature^61, 62^. The presence of SiO_4_ units in the environment of Er denotes the formation of a local atomic structure resembling the one occurring in pyrosilicates^67^ but with an overall wider Er-O-Si bond angle.

## Discussion

**Figure 6:**
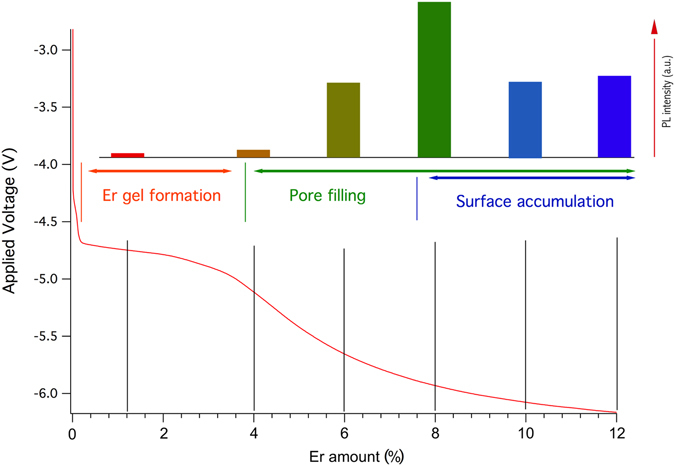
Comparison of the data from the EI and PL as a function of the Er content. The black lines under the PL intensities indicate the amount’s evolution of Er for that sample (the conversion from EI duration and Er content is reported in Table 1). The colored thin vertical lines are drawn to give an approximate indication of where the different EI phases described are present during an EI process. The PL intensities are taken from the Er PL emission data shown in Fig. 2(A) and the EI curve is that reported in Fig. 1.

Figure 6 summarizes most of the experimental results and is intended to facilitate the understanding of the complex phenomena that took place during the different phases of the PSi EI by showing at a glance the different trends emerging by varying the experimental parameters.

In the Figure, the PL data are superimposed to the standard temporal behavior of the EI of the samples in the constant current process adopted in this study. In particular, the EI curve shown in Fig. 6 is the same as that reported in Fig. 1, while the PL intensities are taken from the Er PL peaks of Fig. 2(A).

First, the EI curve reveals that the applied voltage is not constant during the doping process, but shows variations indicating the presence of several EI regimes. As already reported^35–38^, the first double transient observed in the first few seconds of the doping process (EI curve in Fig. 6) has been attributed to the onset of the formation of the Er ethanolate gel during the deposition, which facilitates the Er permanence within the pores due to its higher density with respect to the rest of the solution^37^. The voltage rapidly reaches a quasi-constant state, then its absolute value increases again, suggesting the occurrence of a further variation in the EI process. The quasi-constant voltage state is in agreement with the needle-ET measurements of the PSi_1.2% sample shown in Fig. 4, where an Er accumulation within the pores in form of small nanoclusters that amass near the external surface is apparent. This amassing, although not systematic, leads to an enhanced coverage of the inner pores surface. The following increase instead has already been reported to be likely due to an accumulation of the Er ethanolate gel on the external surface^38^.

In sample PSi_8%_A, most of the pores appear completely filled for a significant portion of their length. Besides, the dark core of the filled pores (needle-ET axial sections reported in Fig. 4, panels H, J, L), strongly suggests that their filling is due to a progressive growth of nanoclusters, starting from the internal surface of the PSi pores and expanding towards their center. This in turn gives rise to the increase of the applied voltage observed for an EI time higher than 100 s, since the observed reduction of the pores effective diameter progressively increases the voltage needed to promote the movement of Er ions into them. The needle-ET of PSi_8%_A sample, displayed in Fig. 4, panels G–L, also clearly indicates that the pores fully filled with Er-rich material are basically morphologically unmodified with respect to those of the untreated sample, while the samples’ surface shows a clearly visible film deposited on the PSi external surface. Coherently with the results reported by Mula *et al*.^38^, where we described the evolution of the superficial Er deposit accumulation given by the formation of the Er ethanolate gel, we observed a progressive macroscopic coverage of the PSi surface in samples PSi_8%_A, PSi_10%_A and PSi_12%_A, starting from the external border of the porous area. We can then roughly identify three diverse regimes in the EI of Er in PSi, indicated in Fig. 6: (A) Er gel formation, (B) pore filling and (C) a concomitant pore filling and superficial film growth. The effect of these different regimes on the PL efficiency can be appreciated if one compares the PL emission intensity to the EI regime reached by the respective PSi:Er samples (Fig. 6). The first, Er-poor samples, show a weak PL emission, while samples with higher Er content (*i*.*e*., in the “pore filling” regime), display a more intense PL signal. The maximum intensity is measured for the PSi_8%_A sample, whose EI time corresponds to the onset of the superficial film deposition. A possible explanation of this is that Er-rich content of the pores has a larger PL emission efficiency with respect to the surface coverage, implying that when the PSi surface is entirely covered with a thin Er-rich deposit the PL shows a slight decrease. The obtained PL results are further supported by the homogenous Er spatial distribution (Supplementary Material Figs S1 and S2) and PL emission, shown in Fig. 2, panels C and D.

Further insights on the samples behavior can be obtained from the measurements of the decay times of the Er-related PL emission (reported in Fig. 2(B)). Sample PSi_6%_A doesn’t show any superficial Er-accumulation, in agreement with the results reported by Mula *et al*.^38^. In fact, as described above, at the corresponding EI time the samples are still in the *pure* “pore filling” regime and below the surface Er-accumulation threshold. The time-resolved PL measurements on PSi_6%_A show two different decay times (τ_1_ = 3.6 µs and τ_2_ = 20 µs). Conversely, for the samples with higher Er content a single shorter decay time (τ ≈ 4 µs) is measured. Such observation may stem from a double origin of the PL emission. The most likely explanation for a double origin is that the thermal annealing of infiltrated PSi samples produces an Er silicate near the pores walls and an Er oxide farther from the inner pores surface, how far depending on the diffusion probability of Si atoms in the Er-rich material. Since we choose a relatively low annealing temperature to prevent a structural modification of the PSi skeleton, this diffusion is expected to be limited. A careful comparison of our results with those from literature for Er oxide^42^ and Er silicate^43^ looks then appropriate. These materials, although emitting PL with similar spectra, typically show significantly different decay times, which may be used as a fingerprint to discriminate what, between the two compounds, is giving rise to the PL signal.

The decay times that were measured are still very far from the millisecond radiative limit of Er emission, and correspondingly the quantum yield is very low. The PL decay time was then taken into account as a quantitative indicator, since nonlinear effects correlated to the excitation level also affect its magnitude, whose analysis is beyond the scope of the present work.

The τ_1_ decay time in PSi_6%_A is almost identical to the single decay time of the other samples and is in the range of those reported by Miritello *et al*.^42^ for Er oxide. In that case, the reported decay time of 8 µs was obtained on samples annealed at 800 °C, and it decreased along with the annealing temperature. If a direct comparison would require a specific detailed analysis, the fact that decay times within the same order of magnitude have been observed suggests that we are measuring photoluminescence from the same compound as Miritello *et al*.^42^. Moreover, these considerations can be extended to the decay time measured on the Er-rich deposition on bulk Si, which is equal to 2.5 µs. The same line of reasoning can be applied when comparing τ_2_ of sample PSi_6%_A with the decay times reported by Miritello *et al*.^43^ for Er silicate, both in the range of tens of microseconds. On the basis of these results and analysis, it looks apparent that the emission from samples having an Er-rich film coverage is mainly from Er oxide, deposited on the samples surface, while the emission from the samples without that superficial coverage mainly comes from the Er silicate inside the porous silicon matrix.

This is further confirmed by the needle-ET results of the sample PSi_8%_A, shown in Fig. 4, panels G-L, and *a fortiori* for the samples with higher Er doping, where the presence of a surface Er-rich fill is clearly observed. Besides, a comparison between the needle-ET results of the PSi_1.2% and PSi_8%_A samples provides further information on the annealing process. Both samples feature well-defined Er-rich regions, with a very limited Er diffusion within the PSi matrix. A better discernment of the Er distribution can be appreciated by the two movies in the supplementary materials, one relating to the PSi_1.2% sample (Movie S1) and the other to the PSi_8%_A one (Movie S2), which display a 3D representation of the data reported in Fig. 4. Thus, while in Movie S1 the Er clusters appear just deposited on the pores walls, in Movie S2 the clusters appear still clearly defined but flattened onto the pores internal surface. On this basis, it is reasonable expecting that, at the interface between the PSi pores walls and the first layer of the deposited Er, the annealing process would lead to the formation of Er silicate. At the same time, the very low presence of Si towards the center of the pores and on the surface of the Er-rich layer, that can be inferred from the very limited interdiffusion of Er and Si observed by the needle-ET imaging, provides a clear indication that both the surface layer and the inner pore content are in fact mainly composed by Si-poor Er oxide. It is worth noting that the accumulation of Er oxide on the sample surface, which is not perfectly homogeneous given the electrochemical doping procedure geometry^38^, also helps explaining the small inhomogeneities of the µPL mapping (Fig. 2(C) and (D)) as due to minor local differences in the surface composition. According to these results, we can infer that the PL emission from highly Er-doped samples (from PSi_8%_A to PSi_12%_A) originates mainly from Er oxide, which is also in agreement with the measured PL decay times for these samples.

On the other hand, in the samples with lower Er doping, the lack of the Er-rich film on the external surface leads to a stronger emission from the Er-doped PSi layer. In this case, the emission comes from both Er silicate (longer decay times, from the regions corresponding to the pores walls) and Er oxide (shorter decay times, from the content of the pores). The increase of the overall PL emission from PSi_6%_A to PSi_8%_A can be explained by the completion of the pores filling process, which implies an increase of the light-emitting material within the porous layer. As mentioned above, further Er deposition seems to reduce the overall PL intensity, even if the data show a new small increase for PSi_12%_A with respect to PSi_10%_A. The first decrease of the PL emission once the surface is covered by the Er oxide film can be explained by arguing that this indicates a larger PL emission from the pores content with respect to the surface film. The observed new PL enhancement with the thickening of the Er surface film with longer Er deposition times can instead be explained by the increase of the amount of the emitting material, which compensates its lesser initial efficiency.

EXAFS data at the Er-L_III_ edge shown in Fig. 5 give additional support to the identification of the luminescent material within the pores as a’local’ Er silicate, probably in a glassy phase. In fact, the observation of both Er-O and Er-Si neighbors resembles what is present in the crystalline silicates (Er_2_Si_2_O_7_^67^, namely) but the absence of higher coordination shells, in particular any RE-RE coordination, suggests the presence of a glassy structure in the long range. Considering that the pore developed surface is about 450 m^2^/cm^3^ ^68^, a dominant fraction of Er ions is in the interface with the Si walls and this is the reason for the observation of the ‘silicate-like’ structure.

## Conclusions

We studied the Er-related photoluminescence emission in porous silicon electrochemically infiltrated with Er to fill the pores with Er-rich materials. A combined analysis of the electrochemical Er infiltration was performed by a multi-technique approach. It showed that the PL emission in Erbium-filled PSi comes from the presence within the pores of Er silicate and Er oxide formed during the electrochemically-driven EI and the following thermal annealing was needed to activate the Er luminescence. From our results, the strongest PL emission from the porous layer is obtained at the end of the “pore filling” phase, where the PL emission comes from both the Er silicate and Er oxide. These results, while unveiling the detailed effects of the electrochemical process, highlight how filling the pores with highly luminescent Er materials instead of finding a compromise between the Er amount and Er clustering in the doping of PSi or SRO structures, could be an effective approach for improving the Er luminescence from Si materials. These results then help identifying where to address the efforts towards highly efficient Er-related emission from porous Si.

## Materials and Methods

### PSi sample preparation and electrochemical infiltration

Porous Si samples were prepared by electrochemical etch in the dark in constant current configuration using a HF:H_2_O:EtOH solution in the 15:15:70 proportion, respectively, following the procedure described by Mula *et al*.^38^.

After fabrication and wash out of the HF solution, PSi samples were put in contact with an ethanolic 0.11 M solution of Er(NO_3_)_3_·5H_2_O salt. The samples were let in contact with the solution for one minute before beginning the electrochemical process. A slow stirring was used to facilitate the solution exchange at the external surface of the PSi layer. This process was performed in constant current mode, using a current density *d*_I_ = 1.6 mA/cm^2^. The duration of the infiltration process determinate the Er amount in the PSi matrix. The EI times were 30, 100, 150, 200, 250 and 300 s to obtain Er amounts of 1.2, 4.0, 6.0, 8.0, 10.0, 12.0%, respectively. Given the gradient in the Er content, these values refer to the Er content towards the external surface.

### Samples thermal treatment

The PSi:Er samples were put under vacuum for 15 hours in the furnace at room temperature before the thermal treatment. Then a 30sccm flux of N_2_ was started and maintained throughout the thermal process. The temperature was then raised to 700 °C and maintained constant for 2 hours. The cooling of the samples was also performed under the gas flux until room temperature was reached.

### Photoluminescence and micro-photoluminescence

Photoluminescence measurements have been performed at room temperature in continuous wave (CW) mode using as excitation the 532 nm light from a diode-pumped solid-state laser. This is the same wavelength successfully used for studying the PL increase in SOI structures codoped with Er and O. Laser excitation power was 770 mW and the spot size about 200 µm. The optical emission from the samples was dispersed with a 30 cm focal length Acton spectrometer equipped with a 75 groves/mm grating blazed at 1200 nm; the detector was a InGaAs Andor line CCD.

For time resolved photoluminescence we employed a pulsed regenerative amplifier as light source (Quantronix Integra, 100 fs pulse duration, 1 KHz repetition rate, 1 mJ energy/pulse, 800 nm in wavelength), while detection occurred with the same spectrometer used for CW measurements, but directing the light to a second output port equipped with a Hamamatsu InGaAs fast photomultiplier. The photomultiplier was used in current mode and its signal detected with a Tektronix GHz oscilloscope.

Micro-photoluminescence (µPL) measurements and mappings were carried at room temperature using a Horiba LabRAM HR spectrometer. A 633 nm laser line was focused with a 100x Leica objective (numerical aperture = 0.9). 2 mW laser power was measured at the sample. The spectrometer was configured with a 150 grooves/mm grating and an InGaAs array. Due to long acquisition times, a background spectrum was systematically subtracted.

### SEM – EDS

The SEM-EDS measurements were performed by using a Zeiss Merlin scanning electron microscope, equipped with a Schottky field emission gun, a Raith pattern generator, an Oxford EDS X-Max SDD detector with an area of 80 mm^2^ and the AzTecEnergy EDS analysis software. The SEM image of the samples surface and that of the PSi layers in cross sectional view were acquired collecting the secondary electron (SE) signal, with the microscope working at an acceleration voltage of 4 kV, a beam current of 300 pA, and by the in-chamber and in-lens SE detector, respectively. To collect both the EDS elemental maps and spectra, the microscope operated at an acceleration voltage of 15 kV, with a beam current of 5.5 nA. The X-Ray peaks chosen for both the EDS mapping and quantitative analysis were the O K, Si K and the Er L, respectively. EDS quantitative analysis was performed by using a ZAF standardless method^58, 69^.

### EXAFS

EXAFS data at the Er-L_III_ edge have been collected at the BM08-LISA (former GILDA)^70, 71^ beamline at the European Synchrotron Radiation Facility. The monochromator was equipped with a pair of Si (311) crystals and was run in dynamically focusing mode. Mirrors coated with Pd were used for beam collimation/focusing and harmonic rejection (E_cutoff_ ≈ 18 keV). The absorption coefficient from the sample was measured in fluorescence mode using a 12 elements array of high purity Ge detectors with an energy resolution of about 200 eV on the Er-L_α_ line. For each sample 2 to 4 spectra were collected and averaged to increase the signal-to-noise ratio. EXAFS spectra were extracted and analyzed with the ATHENA-ARTEMIS codes^72^. Theoretical EXAFS signals were calculated with the Feff8 code^73^ starting from the crystallographic model of Er_2_Si_2_O_7_^67^.

### Electron tomography

ET is based on the collection of a series of Scanning Transmission Electron Microscopy (STEM) images of a sample tilted over a 180° tilt range ideally with an appropriately defined angular step^74^. Needle-ET needs a needle-shaped sample with a diameter of a few hundred nanometers. Needle-shaped samples are prepared using a Focus Ion Beam (FIB) FEI Strata instrument^75^. After depositing protective layers (Tetraethyl orthosilicate (TEOS) followed by W) on the surface of the specimen, a chunk containing the PSi thin film is extracted. The chunk is then glued at the top of a tip, annularly milled by the ion beam to obtain the desired needle-shaped sample geometry and finally inserted in the transmission electron microscope (TEM).

Tilt series acquisition have been performed on a FEI Titan Ultimate TEM (PSi_0% and PSi_1%) and a FEI Titan Themis TEM (PSi_1.2% and PSi_8%_A), working in Scanning (STEM) mode with a High Angle Annular Dark Field (HAADF) detector, with the aim to both enhance the contrast due to the atomic number difference between PSi and erbium and to prevent from most of the diffraction one that could limit a reliability of the following sample volume reconstruction^74^. The STEM has been operated at an acceleration voltage of 80 kV in order to limit as much as possible the beam damage on the PSi samples. Tilt series acquisitions have been composed of 181 images (projections) acquired with a constant tilt step of 1°. Dedicated algorithms have then been used to reconstruct the volume from the acquired images’ series^76^. If compared to the conventional ET, needle-ET permits acquisition over a 180° tilt range due to the needle form of the sample. This ideal case avoids the typical missing wedge artifacts, unavoidably occurring when the imaged samples is prepared in form of lamella or thinned and mounted/deposited on a transmission electron microscopy support. Still, since needle-ET projections are misaligned and tilt axis is not perfectly known, their fine alignment is a fundamental step to be performed prior to any 3D reconstruction. In a recent work^77^ we then proposed a novel and thorough procedure constituted by self-adapting projection denoising, automatic and accurate alignment and determination of tilt axis, and final 3D reconstruction. If compared to previously adopted procedures, this one runs following a more robust and less user dependent routine, leading to high quality 3D reconstruction with fewer artifacts and more reliable results. Finally, to correct the possible sample deformation that may occur during the needle-ET tilt series acquisition a novel non-rigid alignment has been adopted, preventing from deformation-related artifacts in the 3D reconstruction^78^.

The whole procedure reported by Printemps *et al*.^77^ has been applied for projection denoising, alignment and reconstruction of the three samples volume using a Matlab-based custom-made software. Segmentation of the 3 parts (Si, Er and voids) of the 3D volume of the samples has been made by multi-thresholding, exploiting the projection denoising and the following high quality Accelerating voltage used was 80 kV to limit radiation damage on the PSi samples. The videos (Movie S1 and Movie S2) that display a virtual inspection of the segmented volume of PSi sample PSi_1.2% and PSi_8%_A, respectively, have been then created from the volume reconstruction using the Amira Software.

### Data and Materials Availability

All data needed to evaluate the conclusions in the paper are present in the paper and the Supplementary Materials. Additional data related to this paper may be requested from the authors.

## Electronic supplementary material

Supplementary material

Supplementary Movie S1

Supplementary Movie S2
