# Supplementary material for: Doping porous silicon with erbium: pores filling as a method to limit the Er-clustering effects and increasing its light emission
Source: Sci Rep. 2017 Jul 20;7:5957. doi: 10.1038/s41598-017-06567-4 (PMC5519753; doi:10.1038/s41598-017-06567-4)
Supplement: Supplementary file 1 — Supplementary material [file 41598_2017_6567_MOESM1_ESM.pdf]

Supplementary material

Doping porous silicon with erbium: pores filling as a method to limit the Er-clustering effects and increasing its light emission

Guido Mula[1,2], Tony Printemps[3,4], Christophe Licitra[3,4], Elisa Sogne[5], Francesco D'Acapito[6], Narciso Gambacorti[3,4], Nicola Sestu[1], Michele Saba[1], Elisa Pinna[1,2], Daniele Chiriu[1], Pier Carlo Ricci[1], Alberto Casu[5], Francesco Quochi[1], Andrea Mura[1], Giovanni Bongiovanni[1], Andrea Falqui*[5]*

1 - Dipartimento di Fisica, Cittadella Universitaria di Monserrato, Università degli Studi di Cagliari, S.P. 8 km 0.700, 09042 Monserrato (Ca), Italy; guido.mula@unica.it

2 - CNR-IOM - Istituto Officina dei Materiali c/o Laboratorio Materiali Porosi, Dipartimento di Fisica, Università degli Studi di Cagliari, Cittadella Universitaria di Monserrato, S.P. 8, km 0.700 - 09042 Monserrato (Ca) - Italy

3 - Université Grenoble Alpes, F-38000 Grenoble, France

4 - CEA, LETI, MINATEC Campus, F-38054 Grenoble, France.

5 - King Abdullah University of Science and Technology (KAUST), Biological and Environmental Sciences and Engineering (BESE) Division, Nabla Lab, Thuwal 23955-6900, Saudi Arabia andrea.falqui@kaust.edu.sa

6 - CNR-IOM-OGG c/o ESRF, LISA CRG, 71 Av. des Martyrs, F-38043 Grenoble (France)

SEM and SEM-EDS characterization

Fig. S1 (A) shows four different low-magnification secondary electron (SE) SEM images of the external surface of a PSi_8%_A sample and the corresponding EDS elemental maps of Si (blue), O (red) and Er (green) being respectively reported below. The external surface of the PSi layer shows a quite homogenous morphology. As expected, a lower silicon amount is mapped in correspondence of the PSi semicircular layer if compared to the area out of it, where the silicon is still in its pristine, bulk form. Besides, again in correspondence of the semicircular PSi layer, the homogenous presence of both oxygen and erbium is revealed, the former being due to the silicon oxidation related to the fabrication of the PSi layer, the latter as a consequence of the ED. Fig. S1 (B) display a high magnification SE-SEM cross sectional image of Er-doped PSi layer: the measured thickness of the porous layer is about 1.3 μm, and in order to understand how the doping Erbium infiltrated the silicon thin pores the layer has been divided in five areas (with height and width of 230 and 2600 nm, respectively) in which the Er atomic percentage has been measured by SEM-EDS quantitative analysis. A small distance between adjacent areas has been fixed equal to 30 nm, in order to keep separated the collection areas of the X-Rays signal, even in case of small vertical sample drift during the EDS spectra acquisition. Panel (C) finally reports the Er atomic percentage as measured in the five areas reported and numbered in panel (B), where the distance of the five areas from the PSi layer's external surface is measured at their half height and is equal to 115, 345, 575, 805 and 1035 nm, corresponding to areas numbered 1, 2, 3, 4 and 5, respectively. The Er atomic percentage values reported in the graph of panel (C) have been determined using a ZAF standardless quantification method[59] and taking as main elements to be quantified only silicon and erbium, thus fixing equal to 100 the sum of their respective percentages. The about linear decrease of the erbium amount along the PSi layer's depth/formation direction is qualitatively analogous to that measured using the same technique and approach reported in our previous works[35,36]. Even in the present case, the lowering of Er content with the increasing distance from the PSi external surface is a consequence of the PSi columnar structure: the Er amount's decrease within the doping solution is ascribed to its decreasing erbium exchange efficiency along the PSi thin pores depth.

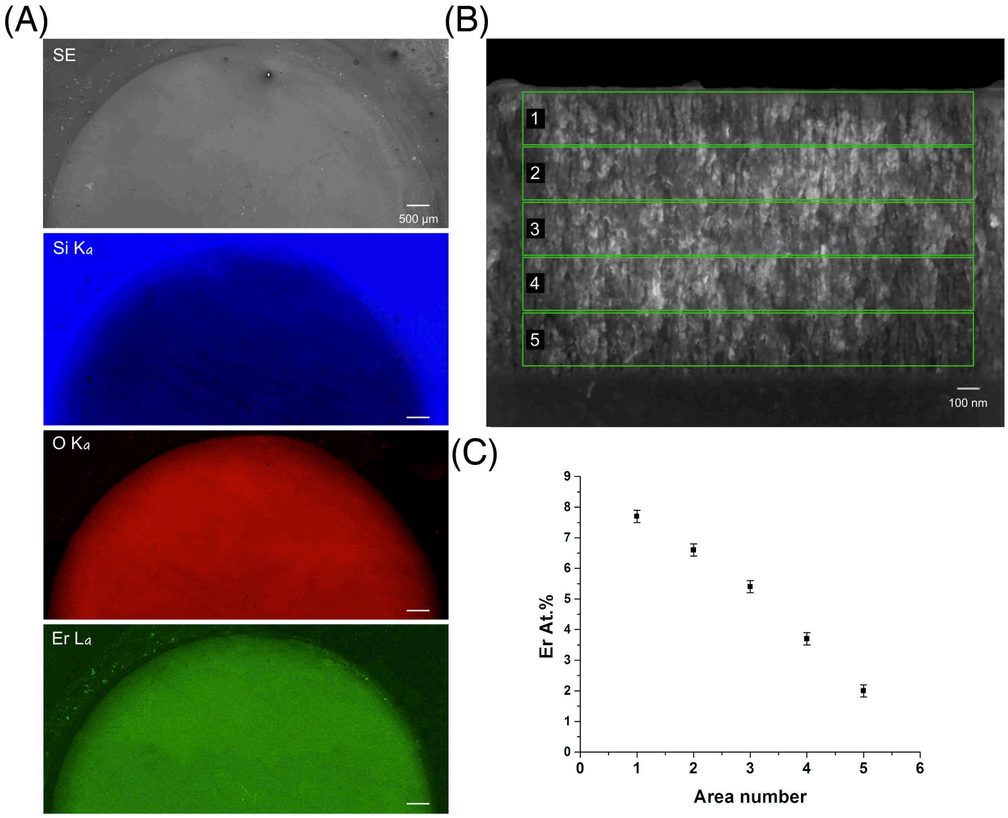

Fig. S1. SEM imaging, EDS elemental mapping and spatially resolved quantification of the Er content of PSi_8%_A sample. *(**A**): SE-SEM low magnification image of the sample's planar view; below the corresponding EDS elemental maps of Si (blue), O (red) and Er (green) are reported, respectively. (**B**) SE-SEM image of the Er-doped PSi layer: the measured thickness of the porous layer is 1.3 µm, divided in five areas (with height and width of 230 and 2600 nm, respectively) in which the Er atomic percentage has been measured by EDS quantitative analysis. (**C**) Er atomic percentage as measured in the five areas reported and numbered in panel (B). The Er atomic percentage values have been calculated simply fixing equal to 100 the sum of Erbium and Silicon respective percentages.*

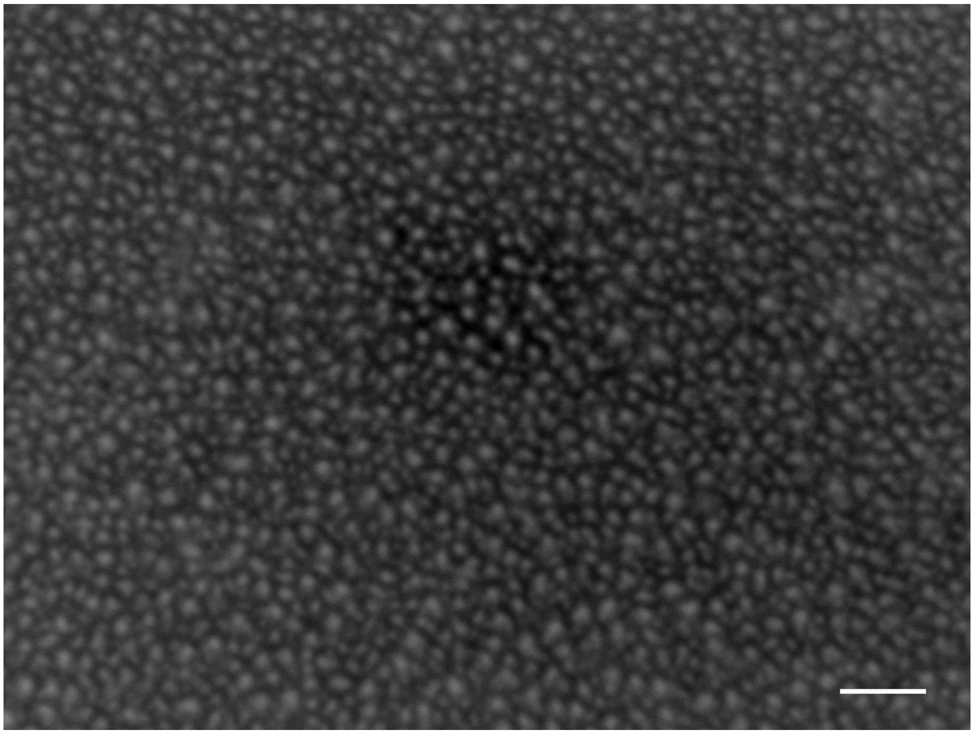

Fig. S2. BSE-SEM image of a PSi_8%_A sample. *The bright spots correspond to the pores filled with Er-rich material.*

The presence of a thin surface layer is visible as a diffused brightness in the sample's surface. This is possible given the collection depth for the SEM signal, greater than the thickness of the Er-rich overlayer.

The darker central area of the image additionally shows a region where there is the presence of some empty pore. Scale bar length: 100nm.

It is interesting to observe a PSi_8%_A sample top view recorded in backscatter configuration (BSE-SEM), shown in Fig. S2. In BSE the higher the atomic number the higher the backscattering efficiency. The small whiter spots on the image are then the signal from the PSi pores filled with Er. A quite homogeneous filling is observed, even if some of the pores appear empty (black)[60,61].

Some additional considerations must be spent on the comparison of SEM-EDS (Fig. 1) and NET measurements (Fig. 4). In EDS elemental mapping, the X-Ray signal coming from the different element is due to the electron penetration in the Er-doped PSi layer. The well-known Kanaya-Okayama equation allows to estimate such a penetration depth, knowing the atomic weight and number, as well as the density of the irradiated flat sample and the electron beam energy[58]. In our case, our sample is constituted by a PSi layer having thickness of 1.3 μm, and density equal to 55%[35] of bulk silicon. Also, such an Er-doped PSi layer is confining on the bottom with bulk silicon. As a first approximation, the presence and related electron scattering effect from the low amount of erbium distributed all over the PSi pores can be neglected, and taking into account the system as roughly constituted by only silicon, by the Kanaya-Okayama equation a 15 keV-electron beam penetration depth in the porous and bulk silicon is estimated being about 56 and 30 μm, respectively, thus indicating that the whole PSi layer is penetrated by electrons. Furthermore, the Anderson-Hasler equation permits to estimate the maximum depth of the volume from which the X-Rays signals corresponding to *a priori* known critical ionization energy is coming, knowing also the density of the X-Ray emitting material and the electron beam energy. To provide this estimate we made use of the same approximation reported above, then neglecting, in the overall material density, the one due to erbium doping. Under this approximation, being the critical ionization energy of the Er L X-line equal to 6.9 keV, the corresponding X-Ray emitting PSi volume's depth has been calculated being equal to 3.5 μm, which is higher than the actual thickness of the PSi layer. In any case, even taking into account the increase in materials density due to the low amount of erbium, this value is still higher than the PSi layer's thickness. This means that the whole porous silicon layer penetrated by the electron beam is emitting the Er L X-Ray line or, to put it differently, the homogenous Er distribution shown in the erbium map reported in Fig. 1 (A) is due to the integration of the erbium L line's X-Ray signal over the entire volume of the doped PSi layer. This well explains the seeming discrepancy between the homogeneous results of EDS-SEM mapping and the inhomogeneous distribution observed by micro-PL and NET characterization.

Fig. S3 shows NET sections from sample PSi_1%. It should be noted that the density of the bright spots along the needle's length and due to the erbium clusters decreases with increasing the distance from the needle area closer to the external surface of the original PSi layer, in agreement with the Erbium content's trend measured by EDS-SEM and shown in Fig. S1 (B)-(C).

Supplementary - Movies Caption

Movie S1 – NET of PSi_1.2% sample*. The movie is made with the same NET data used for Fig. 4 (panels* ***A-F****). The movie starts showing the whole structure, then evidence the position of Er deposition and finally shows the inner pores volumes with and without Er.*

Movie S2 – NET of PSi_8%_A sample*. The movie is made with the same NET data used for Fig. 4 (panels* ***G-L****). The movie starts showing the whole structure, then evidence the position of Er deposition and finally shows the inner pores volumes with and without Er.*

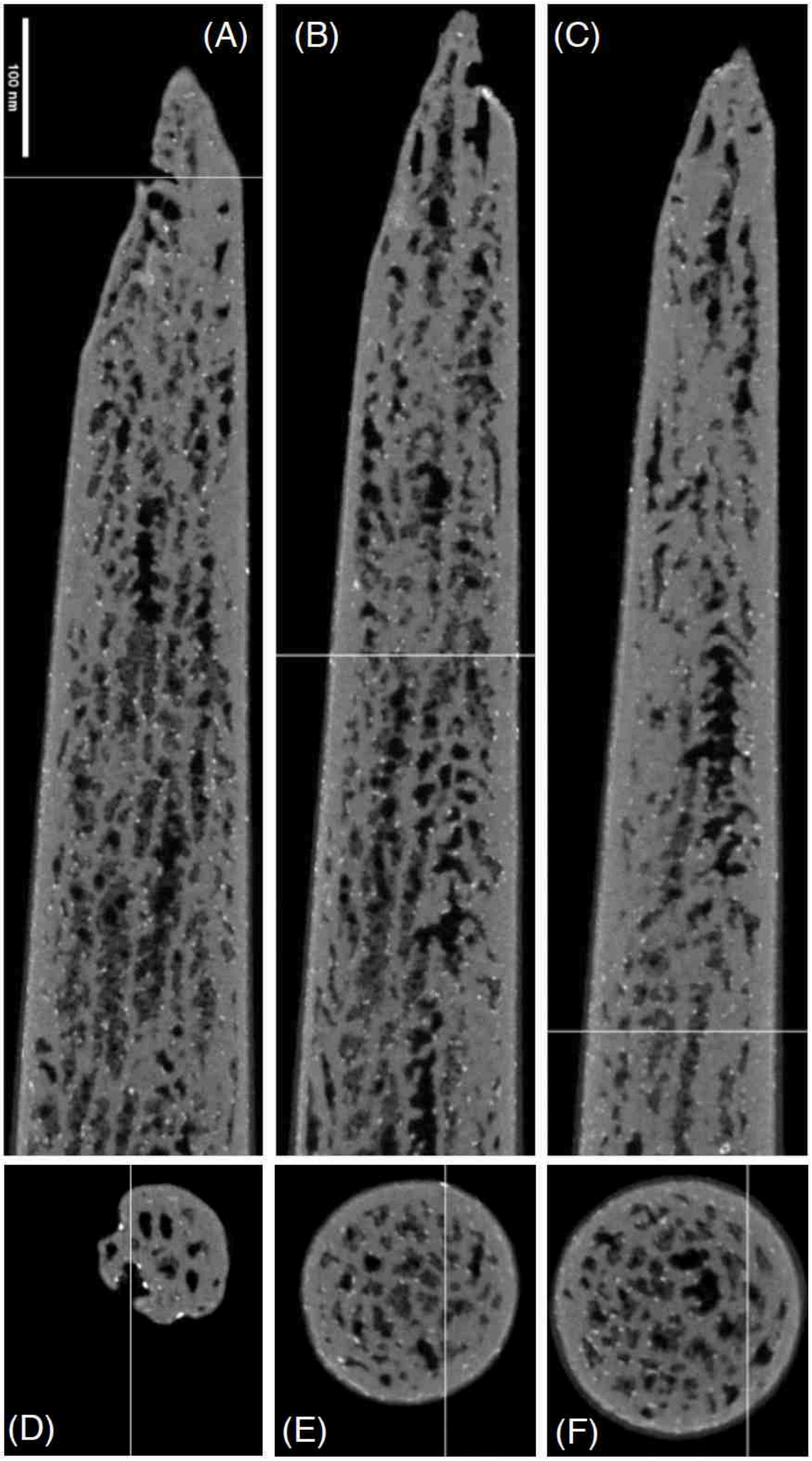

Fig. S3. NET of PSi_1% sample*. The needle was extracted from the middle of the sample. (****A****)-(****C****) three different sagittal sections and (****D****)-(****F****) three different axial sections extracted from the 3D reconstructed volume. Localization of the sagittal sections are represented by a white line on the corresponding axial slices and vice versa. The surface of the PSi specimen is on the top side of the panels A to C.*
